# Chilaiditi syndrome presenting with epigastric pain that improved by intentionally taking the left lateral decubitus position

**DOI:** 10.1002/jgf2.372

**Published:** 2020-09-20

**Authors:** Hitomi Shimada, Masaki Tago, Midori Tokushima, Shu‐ichi Yamashita

**Affiliations:** ^1^ Department of General Medicine Saga University Hospital Saga Japan; ^2^ Shimada Hospital of Medical Corporation Chouseikai Saga Japan

**Keywords:** Chilaiditi syndrome, left lateral decubitus position

## Abstract

Chilaiditi syndrome could improve simply by taking the left lateral decubitus position, avoiding unnecessary hospitalization, or shortening the length of hospital stay. Therefore, repositioning is a noninvasive and effective first‐choice treatment.

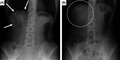

A 44‐year‐old woman presented with squeezing epigastric pain that lasted approximately 30 minutes and developed after eating lunch during her work as a nurse. She also had a history of a similar episode 2 years earlier that improved spontaneously without treatment. On arrival, she was alert, her height was 156 cm, weight: 51 kg, body temperature: 36.6°C, blood pressure: 124/68 mm Hg, pulse rate: 72 beats/min, and percutaneous oxygen saturation: 98% on room air. Her abdomen was soft and flat without tenderness or muscular guarding. Blood test results were unremarkable. Abdominal X‐ray revealed colonic gas immediately below the right diaphragm (Figure 1A). Abdominal computed tomography without enhancement revealed the colon positioned between the anterior thoracic wall and the front edge of the liver, with no other abnormalities (Figure 2). She was diagnosed with Chilaiditi syndrome, for which we recommended taking the left lateral decubitus position as a treatment, and which resulted in sudden disappearance of the pain after 120 minutes. Abdominal X‐ray then showed disappearance of the colonic gas (Figure 1B). Seven months after this episode, she developed similar epigastric pain, with colonic gas seen immediately below the right diaphragm on abdominal X‐ray. We considered this episode a recurrence, and her symptoms improved 30 minutes after taking the left lateral decubitus position.

**FIGURE 1:**
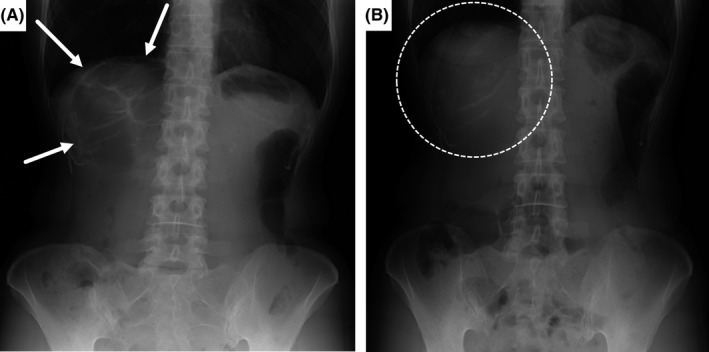
A, Abdominal X‐ray, upright position taken when the patient presented with epigastric pain. The abdominal X‐ray image shows colonic gas immediately below the right diaphragm (arrows). B, Abdominal X‐ray, upright position taken after the disappearance of epigastric pain. The abdominal X‐ray image shows disappearance of the colonic gas immediately below the right diaphragm (dashed circle)

**FIGURE 2:**
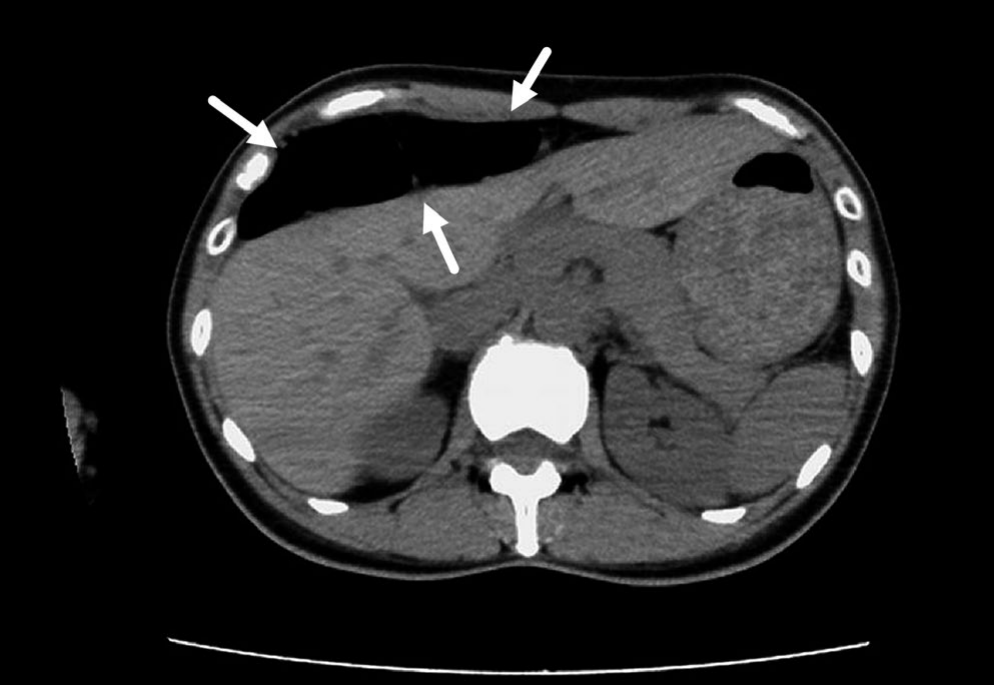
Abdominal computed tomography image without enhancement. The image shows the interposition of the colon between the anterior thoracic wall and the front edge of the liver (arrows), with no other abnormalities in the abdominal cavity

Interposition of the colon between the inferior surface of the diaphragm and the superior surface of the liver is called the Chilaiditi sign, which is usually asymptomatic.^1^ When this sign presents with abdominal symptoms, the condition is called Chilaiditi syndrome.^1^ Although Chilaiditi syndrome usually improves in a few hours or after several days with conservative treatment alone, such as fasting and hydration, and only occasionally requires intestinal decompression,^1^, ^2^, ^3^ some patients require surgical intervention.^1^, ^4^ One reported case is striking because the Chilaiditi sign disappeared after the patient fortuitously took the left lateral decubitus position.^3^ We also recommended that our patient take the left lateral decubitus position as a treatment, which was successful. The suggested mechanism for resolution of signs is that taking the left lateral decubitus position moves intestinal contents to ease flexion at the hepatic flexure, induces the gastrocolic reflex through retention of gastric contents, or facilitates bowel peristalsis through activation of the parasympathetic nervous system, resulting in disengagement of the colon from between the inferior surface of the diaphragm and the superior surface of the liver.^5^ Our patient experienced a similar episode 7 months after the reported episode, and her history of epigastric pain 2 years earlier could also have been an episode of the condition.

To our knowledge, this is the first report of recurrent Chilaiditi syndrome, successfully treated by intentionally taking the left lateral decubitus position. Chilaiditi syndrome could improve simply by taking the left lateral decubitus position, avoiding unnecessary hospitalization, or shortening the length of hospital stay. Therefore, repositioning is a noninvasive and effective first‐choice treatment.

## CONFLICT OF INTEREST

The authors have stated explicitly that there are no conflicts of interest in connection with this article.

## AUTHOR CONTRIBUTION

HS involved in literature search, drafting, and clinical care of the patient. MT: involved in literature search, concept, and drafting. MT: involved in literature search and drafting. SY: involved in concept and revision of article.

## ETHICAL APPROVAL

This manuscript conforms to the provisions of the Declaration of Helsinki in 1995 (as revised in Brazil 2013).

## INFORMED CONSENT

The patient was given informed consent, and patient anonymity was preserved.
